# Monocyte response after colorectal surgery: A prospective cohort study

**DOI:** 10.3389/fimmu.2022.1031216

**Published:** 2022-10-27

**Authors:** Pim P. Edomskis, Willem A. Dik, Cloë L. Sparreboom, Nicole M. A. Nagtzaam, Adrie van Oudenaren, Daniël P. V. Lambrichts, Yves Bayon, Noah N. N. van Dongen, Anand G. Menon, Eelco J. R. de Graaf, Peter Paul L. O. Coene, Johan F. Lange, Pieter J. M. Leenen

**Affiliations:** ^1^ Department of Surgery, Erasmus University Medical Center, Rotterdam, Netherlands; ^2^ Department of Immunology, Erasmus University Medical Center, Rotterdam, Netherlands; ^3^ Laboratory Medical Immunology, Erasmus University Medical Center, Rotterdam, Netherlands; ^4^ Medtronic - Sofradim Production, Trevoux, France; ^5^ University of Amsterdam, Psychological Methods Group, Amsterdam, Netherlands; ^6^ Department of Surgery, IJsselland Ziekenhuis, Capelle aan den IJssel, Netherlands; ^7^ Department of Surgery, Maasstad Ziekenhuis, Rotterdam, Netherlands

**Keywords:** surgery, inflammatory response, monocyte, leukocyte, colorectal carcinoma

## Abstract

**Background:**

Tumor resection is the common approach in patients with colorectal malignancy. Profound insight into inflammatory changes that accompany the normal post-operative stress response will establish reference parameters useful for identification of putative complications. Alterations in circulating monocytes might be indicative as these cells are considered to be the most responsive leukocytes to trauma. Therefore, the aim of this study is to assess the monocyte subset kinetic and phenotypic changes in response to surgery.

**Methods:**

Fifty patients undergoing colorectal tumor resection were included in a multicenter prospective cohort study. Blood samples were collected early in the morning prior to surgery and the next days through postoperative day three for flowcytometric analysis. Leukocyte subtypes were identified and expression of activation stage-related markers by monocyte subsets was quantified.

**Results:**

Changes in leukocyte subset composition and monocyte subset phenotypes were most prominent at the first day postoperatively, after which these parameters typically returned to normal or near-normal preoperative values. The immunophenotypic alterations after surgery were most notable in classical and intermediate monocytes. These included up-regulation of activation markers CD64 and CD62L, but down-regulation of HLA-DR and CD54. Markers of de-activation, CD163 and CD206, were consistently increasingly expressed.

**Discussion/conclusion:**

The current study gives detailed insight into the peripheral blood leukocyte response after colorectal cancer surgery. This form of short-term stress induces a rapid and significant redistribution of immune cells. Immunophenotypic alterations in monocytes as a response to surgery suggest a mixed profile of cellular activation and de-activation.

## Introduction

Colorectal cancer is the third most common cancer worldwide. It is the third most common cancer in men and the second most common cancer in women. There were more than 1.9 million new cases of colorectal cancer in 2020 (1). Surgery is often the main treatment for early-stage colon cancers. After surgery, pro-inflammatory cytokines are normally secreted, a dysregulated release having been associated with postoperative complications (2). Noteworthy complications such as surgical site infection and anastomotic leak (AL) are causes for morbidity, mortality, prolonged length of stay and related costs, and often impaired quality of life (3, 4). Most postoperative complications after colorectal surgery are of infectious origin, and profound insight into inflammatory changes in circulation that accompany the normal post-operative response might help to identify early changes associated with infectious complications (5).

The innate immune system typically becomes activated *via* acute or short-term stress, for example by infection, trauma or surgery, which induces a local tissue response with release of cytokines. Efficient mobilization of leukocytes from storage in spleen and bone marrow into the circulation is crucial for effective host defense. Stress hormones orchestrate a large-scale redistribution of immune cells in the body (6–8). Norepinephrine and epinephrine in particular mobilize immune cells into the bloodstream. Subsequently, epinephrine and cortisol induce emigration from the circulation to tissue surveillance pathways, lymphoid tissues, and sites of ongoing or *de novo* immune activation (9).

Innate immune cells play an important role in tissue defense and repair, even more so when bacterial contamination hardly can be avoided, which is the case in colorectal surgery. For example, sepsis as a major adverse event following complex abdominal surgery is commonly due to bacterial infiltration and can lead to progressive clinical deterioration, organ dysfunction and eventually septic shock with high mortality rates. Upon tissue damage, neutrophilic granulocytes and monocytes enter the tissue from the bloodstream to remove tissue debris and pathogens (10).

The early response after colorectal surgery is characterized by an increase in neutrophils and a decrease in lymphocytes in circulation (11). Monocytes typically show a rapid initial decrease after surgery but then increase compared to preoperative levels (12). This is particularly interesting while monocytes are considered to be the most responsive leukocytes in response to trauma (13). Peripheral blood monocytes can be divided into subsets based on their CD14 and CD16 expression levels (14). Classical monocytes (CD14^++^CD16^-^) develop in the bone marrow from myeloid progenitor cells and enter the circulation where they may differentiate into intermediate monocytes (CD14^++^CD16^+^) and, subsequently, to non-classical monocytes(CD14^+^CD16^++^) (14). Classical monocytes represent the most prevalent subset in peripheral blood and are prime responders to inflammatory stimuli. Intermediate monocytes are the most potent producers of pro-inflammatory cytokines, whereas non-classical monocytes patrol blood vessels and are generally considered to be anti-inflammatory (15).

Extensive immunophenotyping of peripheral blood immune cell populations has proven to be a good way to track leukocyte kinetics after surgical trauma, but this has only been tested in hip surgery, which is basically a sterile environment (12). To our knowledge there is no study with in depth evaluation of monocyte subset distribution and phenotype in peripheral blood in relation to colorectal cancer surgery.

To further improve insight into the normal post-operative course after colorectal surgery, the aim of this prospective study is to assess the monocyte subset kinetics and phenotypic changes. This is performed during the first three days postoperatively and compared with the preoperative state. In this, we focus on immunophenotypic indicators of immunosuppression as well as inflammatory activation as both conditions are known to occur after surgery (16–18).

## Materials and methods

### Study design and patient population

This study was designed as a multicenter prospective cohort study. Three hospitals in the South-West region of the Netherlands participated in the study. Patients were included between August 2017 and November 2019. Treatment-naïve patients diagnosed with colorectal carcinoma, aged 18 years and above who underwent colorectal resection (either right hemicolectomy, left hemicolectomy, sigmoidectomy or low anterior resection) with construction of an anastomosis were eligible for inclusion. Exclusion criteria were pregnancy, preoperative chemotherapy and/or radiotherapy, inflammatory diseases and regular use of immunosuppressant drugs. It was the intention to perform all operations by laparoscopy. The construction of anastomoses was performed manually or with a stapler; the choice of methodology and the anastomotic configuration were left to the surgeons’ discretion. The here-described research was performed in accordance with the World Medical Association Declaration of Helsinki – Ethical Principles for Medical Research Involving Human Subjects. Thus all patients gave written informed consent prior to the operation and the medical ethical committee of Erasmus University Medical Center in the Netherlands approved this study(NL59261.078.16). Local ethical approval was also obtained in the other participating hospitals. This study was registered at https://www.trialregister.nl/ (study ID NL7369).

### Clinical data assessment

Baseline characteristics (age, sex, body mass index, medication use, bowel preparation, smoking, alcohol use, American Society Anesthesiologists score (19), previous abdominal surgery) were obtained preoperatively. The clinical follow-up ended at 30 days postoperatively.

### Blood sample collection and analysis

Blood samples from participants in the Immune Monitoring After Colorectal Surgery (IMACS) trial were obtained in Vacutainer^®^ venous blood collection tubes with spray-dried K2EDTA (Becton Dickinson, Franklin Lakes, NJ, USA). Blood samples of 10 mL before surgery and 3 mL daily at the first three days after surgery were required. Blood was drawn early in the morning prior to surgery (day 0) and circa every 24 hours up to postoperative day (POD) three. White blood cell count was determined using an automated cell counter (Countess II; Thermofisher, Waltham, MA, USA or Sysmex XP-300; Norderstedt, Germany). Samples were taken for whole blood flowcytometric immunophenotyping for the different monocyte subpopulations as indicated below.

### Flow cytometry

Flow cytometric immunophenotyping of leukocyte subsets was conducted using standard protocols on fresh whole blood samples (80 μL). After labeling, erythrocytes were lysed using NH_4_Cl or BD lysing solution (Becton Dickinson) according to manufacturer’s instructions. Samples were measured on a FACS Canto II (Becton Dickinson) flow cytometer and analyzed using Infinicyt software (Infinicyt 2.0; Cytognos S.L., Salamanca, Spain). The following antibodies were used: CD45, CD66b, CD64, CD14, CD16, CD11b, anti-HLA-DR, CD54, CD62L, anti-TLR4, anti-TLR5, CD163, CD121b and CD206 (specific information in **Supplementary Table 1**). The gating strategy to identify different leukocyte subsets is shown in **Supplementary Figure 1**.

The following monocyte populations were defined, classical monocytes: CD45^+^/CD14^++^/CD16^-^/CD64^+^/CD66b^-^, intermediate monocytes: CD45^+^/CD14^++^/CD16^+^/CD64^+^/CD66b^-^, non-classical monocytes: CD45^++^/CD14^++^/CD16^++^/CD64^+^/CD66b^-^ and dendritic cells CD45^+^/CD14^-^/CD16^-^ (**Supplementary Figure 1**). Further leukocyte populations defined were neutrophils (CD45^+^/CD66b^+^/CD64^-^/CD16^+^), eosinophils (CD45^+^/CD66b^+^/CD64^-^/CD16^-^) and lymphocytes (CD45^+^/CD66b^-^/CD64^-^). For interpretation of monocyte phenotypic changes after surgery, markers were divided into indicators of cellular activation (i.e. generally up-regulated by e.g. LPS, IL-1 or IFN-γ) or cellular de-activation (i.e. up-regulated by anti-inflammatory mediators such as IL-10 or glucocorticoids). Monocyte activation markers defined in this sense are CD64, CD14, CD11b, HLA-DR, CD54, CD62L, TLR4 and TLR5, while CD163, CD121b and CD206 are indicators of monocyte de-activation.

### Statistical analysis

Normal distribution of data was verified by the Shapiro-Wilk test, visualization of the respective histograms and calculation of the Z-score of skewness and kurtosis. The assumption of homogeneity of variances was assessed by the nonparametric Levene’s test. Not normally distributed data consisting of multiple groups were analyzed with the Kruskal–Wallis test and a *post-hoc* analysis using the Bonferroni approach.

Continuous variables that were not normally distributed were described as median ± interquartile range (IQR) and compared with the Mann–Whitney U-test. Categorical variables were described as percentages and compared with the chi-square test or Fisher’s exact test when needed. Correlation analyses were performed using Spearman’s rank correlation. Two-sided p-values ≤ 0.05 were considered statistically significant.

Statistical analysis was performed using SPSS version 27.0 (IBM, Armonk, USA) and Graphpad Prism version 5.0 (GraphPad Software, San Diego, USA).

## Results

### Patient baseline characteristics

To study in detail the stress response and ensuing systemic inflammation following colorectal surgery, the changes in patients’ circulating leukocyte composition and phenotype with specific focus on alterations in monocyte subsets were monitored. Sixty patients undergoing colorectal resection with planned primary anastomosis for colorectal malignancy were approached for study participation. From these, five patients were excluded due to an adapted surgical procedure without creation of an anastomosis and five patients had previously received radio- or chemotherapy and were therefore excluded. Hence, 50 patients were effectively included. A summary of all baseline demographics of the study population of 50 patients is shown in **Table 1**. Every procedure was approached laparoscopically but nine of them were converted to an open procedure, based on surgical considerations.

**Table 1:** Patient characteristics.

	Total no. of patients (n=50)	Missing data
*Patient characteristics*		
Age (years), median ± IQR	66.8 (58.0-75.0)	–
Sex		
Female	26 52.0%	–
Male	24 48.0%	
BMI, median ± IQR	26.4 (23.5-28.3)	–
Smoking^1^	20 40.0%	–
Alcohol abuse^2^	7 14.0%	–
Bowel preparation^3^	16 32.0%	2	4.0%
Corticosteroids^4^	6 12.0%	–
NSAIDS^5^	1 2.0%	–
ASA score^6^		
II	32 64.0%	–
III	18 36.0%	–
*Surgical characteristics*		–
Procedure		
Right hemicolectomy	32 64.0%	
Left hemicolectomy	4 8.0%	
Sigmoid resection	7 14.0%	
Low anterior resection	7 14.0%	
Surgical technique		–
Open	0	
Laparoscopic	50 100%	
Conversion^7^	9 18.0%	
Construction of anastomosis		–
Manual	8 16.0%	
Stapler	42 84.0%	
Configuration of anastomosis		–
Side-to-side	34 68.0%	
Side-to-end	12 24.0%	
End-to-end	4 8.0%	
Diverting ileostomy	2 4.0%	–	
Surgical time (min), median ± IQR	134 (101.3-147.5)	–	

ASA, American Society of Anesthesiologists; BMI, body mass index; IQR, interquartile range. ^1^ Smoking: This group contained current smokers and/or had a history of smoking. ^2^ Alcohol abuse: contained heavy drinkers who drink ≥3 consumptions on average a day. ^3^ Bowel preparation: preoperative oral suspensions to clean the bowel. ^4^ Corticosteroids: those who used corticosteroids on a regular base preoperatively (e.g. asthma inhaler, prednisone). ^5^ NSAIDs: those who used NSAIDs on a regular base preoperatively. ^6^ ASA score: Preoperative screening contains this classification for perioperative risk management. ^7^ Conversion: All procedures were initially done laparoscopically, reasons for conversions were: intra-abdominal adhesions, extensive tumor growth, infectious infiltrate.

### Colorectal surgical stress induces neutrophil, monocyte and DC increase in circulation, but eosinophil decline

**Figure 1:**
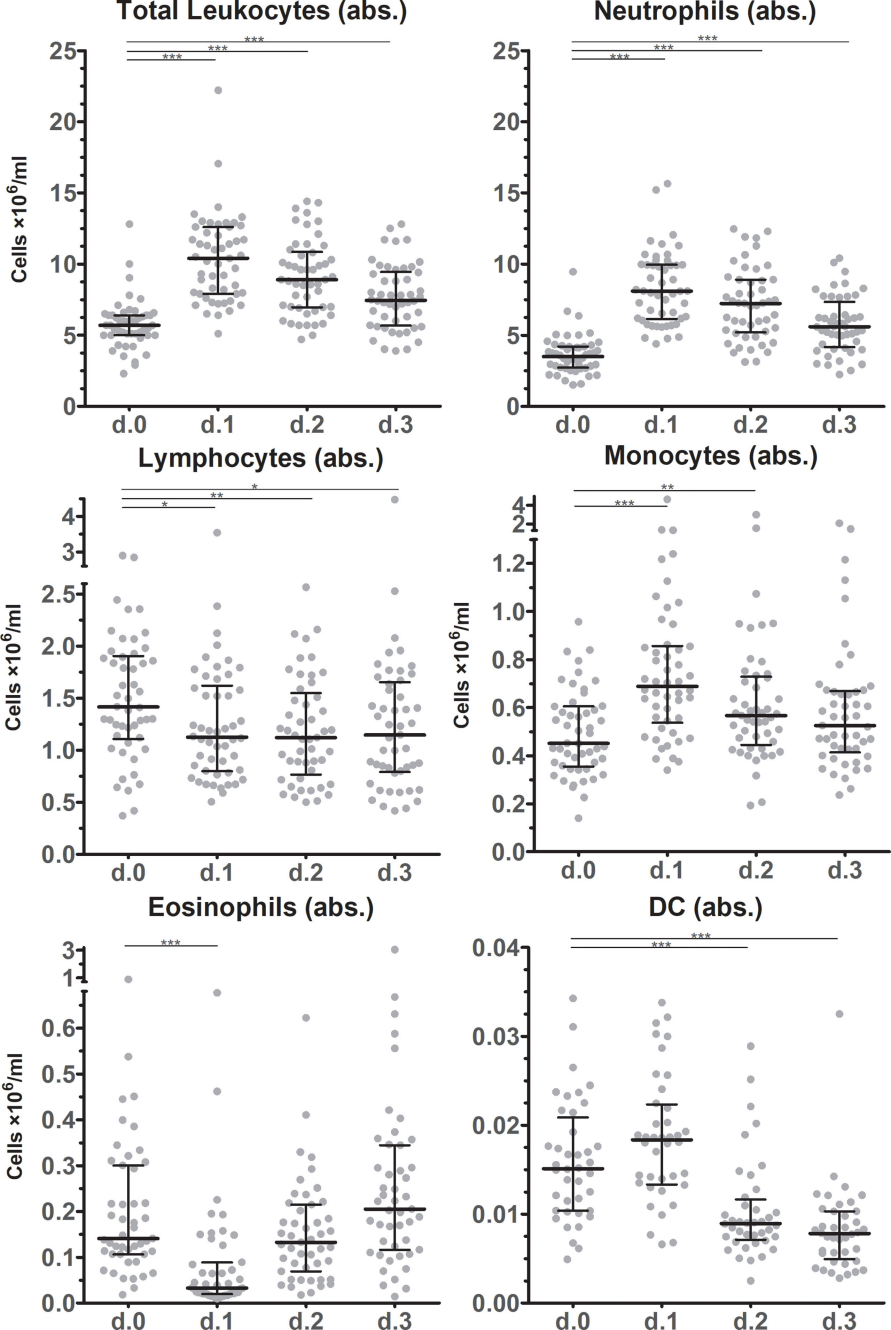
Absolute leukocyte subset counts after surgery. Absolute cell counts of leukocyte subsets in circulation before and during the first three days after surgery as determined by flowcytometric immunophenotyping. Individual data points as well as medians and interquartile ranges are shown. Significant differences between d.0 and d.1, d.2 or d.3 are indicated: * p ≤ 0.05; ** 0.01 > p < 0.001; *** p ≤ 0.001.

**Figure 2:**
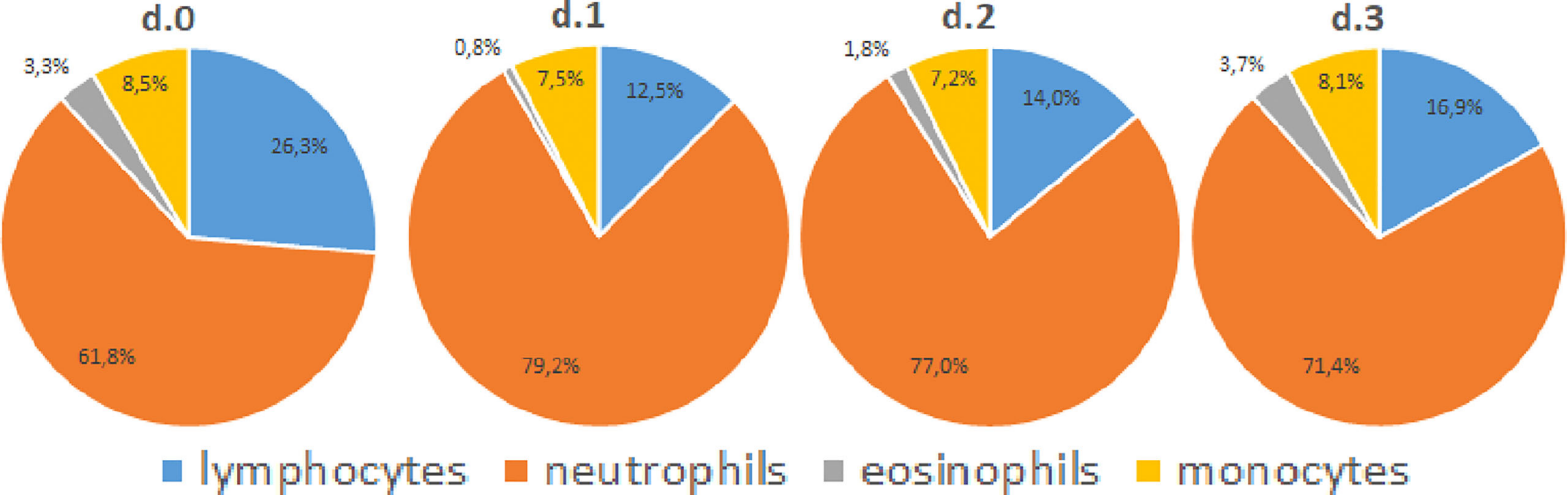
Relative leukocyte subset counts. Pie charts of relative leukocyte cell counts in circulation before and during the first three days after surgery. Medium values are indicated as percentages.

Absolute total leukocyte counts in peripheral blood doubled on average from POD0 to the first postoperative day (POD1) (p < 0.001), and trended to normalize during the following days (**Figure 1**). However, total leukocyte counts were still elevated at POD3 compared to POD0 (p < 0.001). Neutrophils kinetics paralleled the total leukocyte profile at all examined time points. Also total numbers of monocytes increased in circulation from POD0 to POD1. Monocyte numbers returned to preoperative levels at POD3, while DC numbers rose only non-significantly from POD0 to POD1, but significantly declined at POD2 and POD3 to approximately half of the preoperative level (p < 0.001). Absolute lymphocyte counts decreased slightly from POD0 to POD1, and then stabilized through POD3. Finally, absolute eosinophil counts strongly declined from POD0 to POD1 but then rapidly recovered from POD2 onwards to levels at least as high as POD0. On a relative level (**Figure 2**) neutrophils increased in percentage to such an extent that they accounted for more than 75% on POD1, but they deflected towards preoperative levels on the following days. Interestingly, monocytes increased in absolute numbers during the first days after surgery but were remarkably stable in frequency over all days. Lymphocytes and eosinophils declined most from 26.3% (POD0) to 12.5% (POD1) and from 3.3% (POD0) to 0.8% (POD1), respectively.

### Classical and intermediate monocytes subsets show the most dynamic changes after surgery

**Figure 3:**
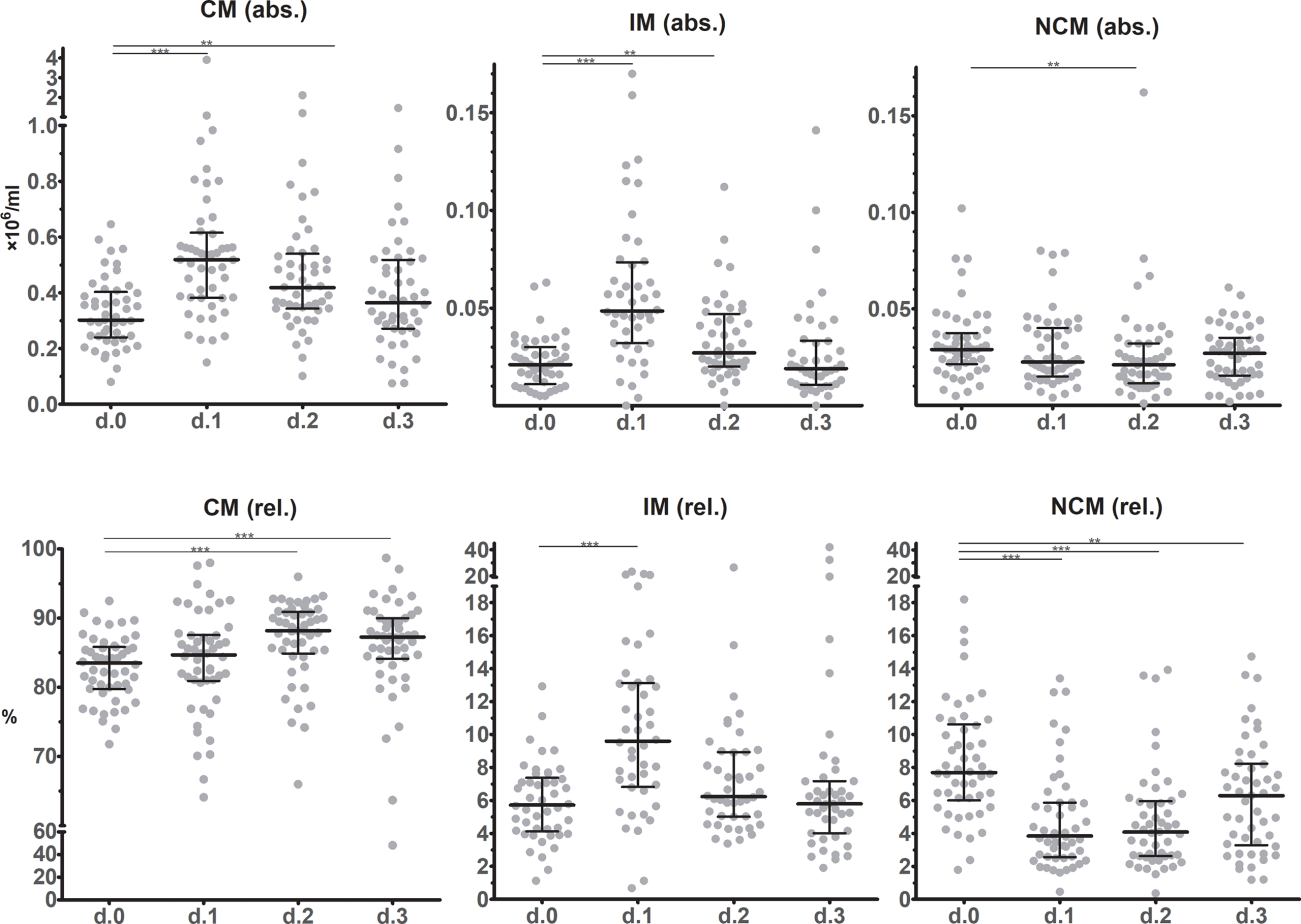
Monocyte subset counts in circulation before and during the first three days after surgery as determined using flow cytometric immunophenotyping. The upper row shows absolute values, while the lower row show frequencies. Individual data points as well as medians and interquartile ranges are shown. CM – classical monocytes; IM – intermediate monocytes; NCM – non-classical monocytes. Significant differences between d.0 and d.1, d.2 or d.3 are indicated: * p ≤ 0.05; ** 0.01 > p < 0.001; *** p ≤ 0.001.

Absolute classical monocyte (CM, CD14^++^CD16^-^) and intermediate monocyte (IM, CD14^++^16^+^) counts showed similar dynamic changes after surgery as they approximately doubled from POD0 to POD1 (**Figure 3**). At POD2 CM and IM counts declined again, reaching preoperative counts at POD3. Absolute numbers of non-classical monocytes (NCM, CD14^+^16^++^) slightly decreased at POD1 and especially at POD2 (P=0.0078) after which they returned to preoperative levels on POD3 again. In relative terms, CM were most abundant (>80% of the monocyte population at baseline). From POD1 up until POD3 CM percentages increased and remained elevated compared to the preoperative state. IM frequencies significantly increased at POD1, whereas NCM declined significantly. During the next days, IM and NCM frequencies returned to pre-operative or near-pre-operative levels.

### Expression of markers indicative of monocyte activation

**Figure 4:**
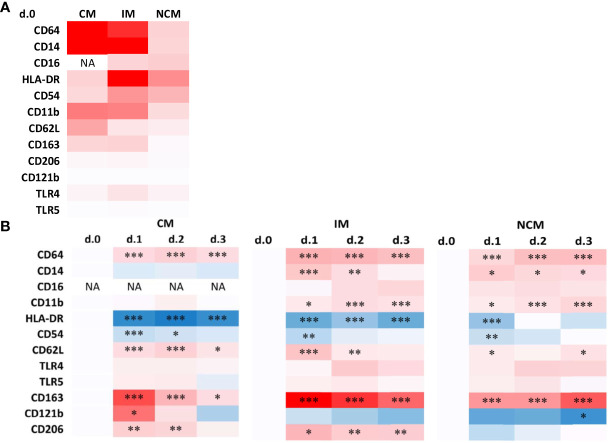
Heatmaps of immunophenotypic marker expression by monocyte subsets preoperatively **(A)** and changes in marker expression during the days after surgery **(B)**. **(A)** Data are presented as a heatmap using the average median fluorescence intensities as determined in all patients. The intensity of red represents the median phenotypic marker expression at POD0. Phenotpic data representing individual patient values are shown in **Supplementary Figure 2**. **(B)** Data are presented as heatmaps of the N-fold increase in median fluorescence intensities for postoperative days compared to POD0. Blue and red color intensity represent down- and upregulation compared to the preoperative baseline levels, respectively. Significant differences between d.0 and d.1, d.2 or d.3 are indicated: * p ≤ 0.05; ** 0.01 > p < 0.001; *** p ≤ 0.001.

It is reasonable to expect that the quantitative changes in monocyte subset composition, induced by the surgical stress response, also are reflected in immunophenotypic alterations indicative of the exposure to mediators released in circulation. Besides differences in CD14 and CD16 expression, which formed the basis of monocyte subset distinction, also other immunophenotypic markers are differentially expressed at POD0 between CM, IM and NCM (**Figure 4A** and **Supplementary Figure 2**).

In immediate response to surgery, the expression of several activation stage-related surface markers showed profound alterations in the different monocyte subsets as visualized in heatmaps using pre-operative values as points of reference (**Figure 4B** and **Supplementary Figure 2**). Overall, changes compared to preoperative levels were most evident at POD1, after which phenotypic trends typically returned to normal or near-normal preoperative values.

In CM, the activation markers CD64 and CD62L showed increased expression on POD1, while HLA-DR and CD54 declined. Markers of de-activation CD163, CD121b and CD206, all significantly increased on POD1 in CM. In general, IM showed similar changes compared to CM as well as an additional increase in expression of activation markers CD14 and CD11b.

NCM showed less profound immunophenotypic changes upon surgery, in accordance with their lack of quantitative response. Changes that did reach statistical significance (**Supplementary Figure 2**) concerned similar changes as observed in the other subsets, such as increases in CD64, CD14 and CD11b, and decreases in HLA-DR and CD54 expression. However most of the markers studied are expressed at a much lower level by NCM than by the other subsets, and changes are relatively marginal compared to adaptations in other subsets.

### Association between patient and surgical characteristics and leukocyte subset dynamics

In this section patient and surgical characteristics and their relation to the short-term leukocyte response between POD0 and POD1 are considered (**Table 2**). The most significant association appeared to be a positive correlation between IM counts and operation time (r= 0.494; p=0.001). Furthermore, we observed a significant negative correlation between male sex and changes in lymphocyte counts between POD0 and POD1 (r= -0.298; p=0.035). This was not found in females. Lastly, we found a negative correlation between age and change in CM counts (r= -0.385; p=0.006) (**Supplementary Figure 3**). This similarly reflected in total monocyte counts. For ASA classification, as measure of general physical status, no significant correlations were found with leukocyte responses.

**Table 2:** Leukocyte subsets and their correlation with clinical parameters d.1 minus d.0.

	Sex^b^	Age (y)^b^	ASA (II VS lll)^a^	OR-time^b^	BMi^b^
Total Leukocytes	NS^c^	NS	NS	NS	NS
Neutrophils	NS	NS	NS	NS	NS
Eosinophils	NS	NS	NS	NS	NS
Dendritic cells	NS	NS	NS	NS	NS
Lymphocytes	M=-0.298; p=0.035; F=NS^d,e^	NS	NS	NS	NS
Total Monocytes	NS	-0.363; p=0.010	NS	NS	NS
Classical monocytes	NS	-0.385; p=0.006	NS	NS	NS
Intermediate monocytes	NS	NS	NS	0.494; p=0.001	NS
Non-classical monocytes	NS	NS	NS	NS	NS

a Mann-Whitney U test (N and p-value).

b Spearman's correlation (r and p-value).^c^NS, not significant; ^d^M, male; F, female.^e^Individual data points shown in **Supplementary Figure 5**.Correlations between changes in leukocyte subsets (d.0 – d.1) and clinical parameters.

**Figure 5:**
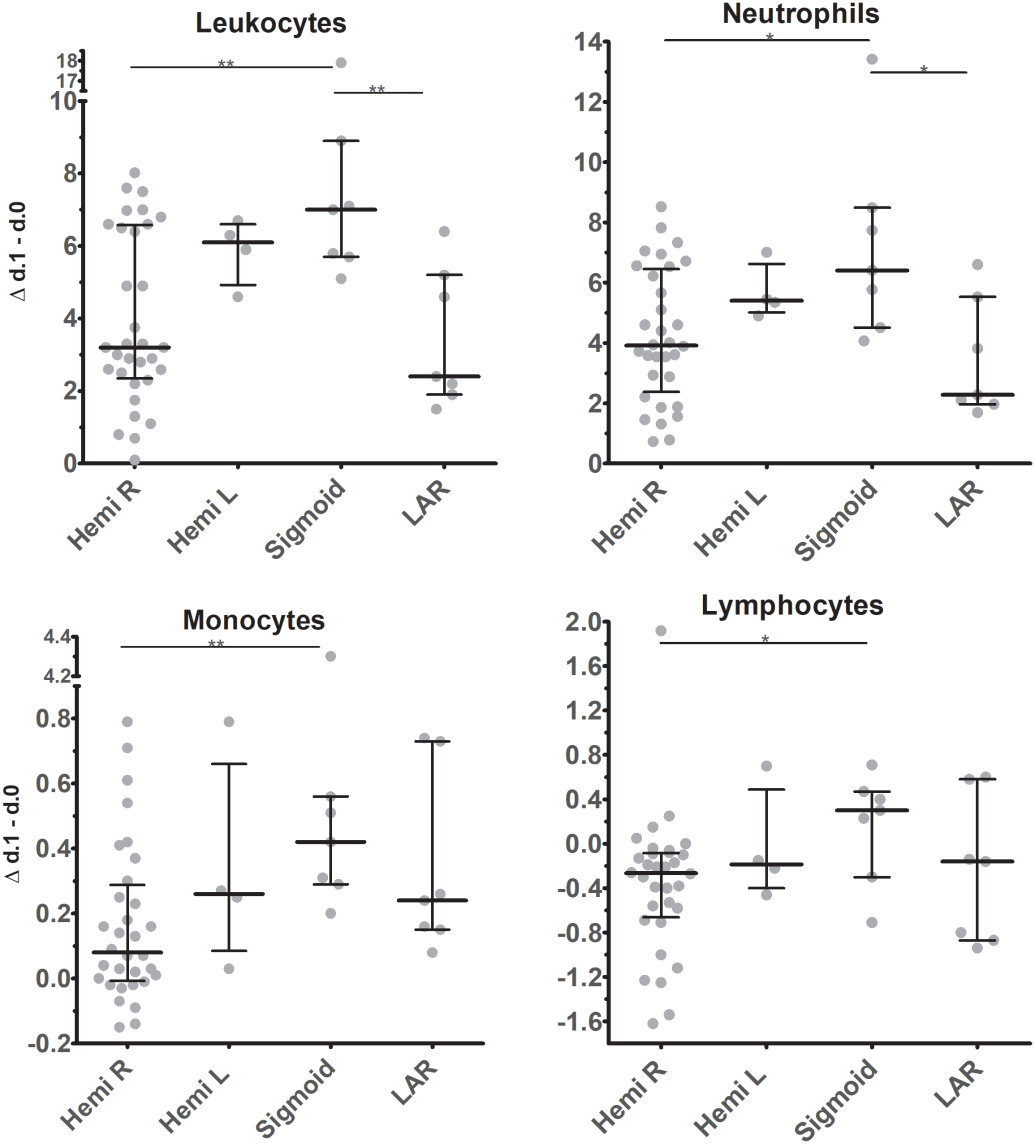
Changes in absolute cell counts of leukocyte subsets in circulation between d.0 and d.1 after surgery in patients undergoing tumor resection at different locations. Individual data points are represented, as are medians and interquartile ranges. For statistics independent Kruskal-Wallis test, with Bonferroni correction was used. Significant differences are indicated: * p ≤ 0.05; ** 0.01 > p < 0.001. Hemi R / L, Hemicolectomy right / left; Sigmoid, Sigmoid resection; LAR, low-anterior resection.

Depending on the location of the tumor, most patients underwent right hemicolectomy (n=32), while a small number of patients underwent left hemicolectomy (n=4), sigmoid resection (n=7), or low anterior resection (n=7). These small numbers warn against over-interpretation, but we deem our results on the differences observed between patient groups of interest as they suggest that the corresponding operation might have significantly different effects on total leukocyte mobilization immediately after surgery (**Figure 5**). Specifically, overall mobilization of leukocytes in peripheral blood was highest in patients who underwent sigmoid resection and lowest after right hemicolectomy and low-anterior resection. In the latter patient populations, differences in response between individual patients were present, while patients undergoing left hemi-colectomy or sigmoid resection all showed high levels of leukocyte mobilization. These differences are reflected in particular in neutrophil and monocyte mobilization, and they are statistically significant in several of the comparisons.

## Discussion

In this prospective study we aimed to get insight into the leukocyte kinetics after colorectal surgery. In particular, we focused on the quantitative and phenotypic changes in distinct subsets of peripheral blood monocytes during the first three days postoperatively as indicators of the cellular response to the acute inflammatory conditions imposed by the surgical procedures.

Absolute total leukocyte counts in blood double from preoperative to the first postoperative day and tend to normalize in the following days. Neutrophils and monocytes increase most after surgery, whereas eosinophils decrease strongly and lymphocyte numbers remain stable. Considering monocyte subsets, absolute CM and IM numbers double from POD0 to POD1 followed and subsequently trend towards the preoperative baseline. In immediate response to surgery, the expression of several surface markers, indicative of cellular activation or de-activation, shows profound alterations in the different monocyte subsets, displaying signs of stimulation into both directions.

Surgery represents acute or short-term stress that typically induces a rapid and significant redistribution of immune cells to sites of damage leading to immune activation. Simultaneously surgery is known to be associated with postoperative immune suppression (16–18). The latter may have important consequences as previously it has been related to infectious complications (20), yet little is known about how to interpret and monitor leukocyte dynamics. Stress-induced leukocyte mobilization and redistribution may be a fundamental survival response that directs leukocyte subpopulations to specific target organs, and significantly enhances the speed, efficacy and regulation of an immune response (9, 21). Stress signals are communicated by direct innervation to the adrenal medulla to cause a nearly immediate release of catecholamines (epinephrine and norepinephrine) and through neuro-hormonal signals *via* the hypothalamic-pituitary-adrenal (HPA) axis that mediate the release of cortisol. Additionally, proinflammatory cytokines (e.g. IL-1, TNF and IL-6) are released upon detection of local damage, and these stimulate production of acute phase proteins such as C-reactive protein, and lead to altered leukocyte immunophenotype and functionality (22). Effectively, leukocyte numbers in circulation are a net result of mobilization from storage in bone marrow and spleen by hormonal and neuronal signals, as well as migration and relocation to the trauma site.

In the present study a rapid increase of neutrophil and monocyte numbers in circulation, a major early disappearance of eosinophils and limited change in lymphocyte numbers were observed. Our current findings on the dynamics of leukocyte subsets in circulation after surgery are in line with and extend previous findings by others (12). Neutrophils and monocytes are mobilized in circulation in response to catecholamine release (21), and increased CXCR2- and CCR2-mediated signals (23, 24). Neutrophil and monocyte numbers change most strongly in response to surgical stress from POD0 to POD1, which can be explained by the above mentioned direct release of mediators for cellular activation and an anti-inflammatory response mediated in particular by glucocorticoids. Total monocyte numbers increase initially but return to preoperative levels at POD3, probably as indicator that the cellular balance is being restored. Acute eosinopenia has already long ago been recognized as a consequence of corticosteroid release (25, 26). The observed acute decrease of eosinophils at POD1 in our study endorses this mechanism.

Lymphocyte mobilization is relatively insensitive to surgical stress, but we observed a remarkable difference between males and females in lymphocyte response. In our study we observed a diminution in circulating lymphocyte numbers from POD0 to POD1 in men, but not women, undergoing surgery. Wichmann et al. previously found a similar male-specific decrease in lymphocytes from POD0 to POD1 after abdominal surgery, in agreement with our finding (27). Although the exact value of this sex difference should be interpreted with caution, it might point to an immunological advantage in women during the early postoperative period after abdominal surgery. This contributes to understanding the difference for women having a better overall survival and colorectal cancer-specific survival (28).

Interestingly, in our study also differences in operation type to be associated with leukocyte mobilization were found. In particular, the difference between the group receiving a right hemicolectomy and a sigmoid resection was striking as there was a significant difference in overall leukocyte, neutrophil, monocyte and lymphocyte number from POD0 to POD1 between these patient groups. A clear-cut explanation of these findings is lacking, but we suggest that the specific location of surgery might influence the release of mediators that determine leukocyte mobilization. Moreover right-sided colorectal tumors are commonly microsatellite-instable tumors, whereas left-sided tumors are more often chromosomal-instable (29). As a result, prior effects of specific tumor types on leukocyte mobilization mechanisms might be another explanation for the difference found.

### Monocyte subsets and markers

Monocytes are considered to be the most responsive leukocytes in response to trauma (13). Immediate response to surgical stress is therefore expected. Monocytes are recruited following tissue alterations from the bone marrow or the marginating pool associated with blood vessels. Subsets of monocytes differ in gene expression and cytokine production, antigen processing and presentation, as well as the capacity for inducing angiogenesis (30). Developmentally they are connected by maturation sequence: CM give rise to IM and subsequently to NCM (31, 32). A shift in monocyte distribution from CM to IM and NCM has already been observed for patients with cardiometabolic disorders, and is generally associated with a chronically inflamed condition (33). An increase in circulating CM numbers after surgery, which preferentially occurs *via* interaction of CCL2 and CCL7 with the chemokine receptor CCR2 (34), was observed in this study. This response has been reported in several studies (12, 35). Also, we found a negative correlation between age and CM number (POD1-POD0) (r=-0.385; p=0.006). This indicates a decreasing ability with age to mobilize classical monocytes, possibly making older patients more vulnerable to infectious complications (36). Although this may not be surprising in view of decline in immune function upon aging, it is remarkable that we only found this association for classical monocytes, and not for neutrophils.

Circulating IM showed an increase in our study at POD1 compared to POD0. Another monocyte subset study by van den Bossche et al. is in line with our study and found similar kinetics. However, they found an additional early decrease directly after surgery followed by an increase at 24 hours after surgery (12). This phenomenon can be explained by the (predominant) association of IM and NCM with the vascular wall and the mobilization from the marginating pool after exposure to catecholamines that were released as a result of perioperative stress. In a study with patients undergoing elective cardiac surgery direct postoperative elevated IM was a predictive marker for extracardiac complications (35), possibly as a result of increased stress perioperatively. Also, we found IM number and operation time to be positively correlated (r=0.494; P=0.001). The latter could be due to prolonged release of stress mediators (e.g. catecholamines), which can result in a pro-inflammatory response with increased mobilization of monocytes from the marginating pool. IM typically are major producers of pro-inflammatory cytokines, therefore this increase is in line with this thought.

In order to analyze monocyte responses to surgery at a more detailed level, application of phenotypic markers can help as their expression levels characterize differentially activated monocyte stages. In general, the observed immunophenotypic alterations in monocytes upon surgery are suggestive of a mixed profile of cellular activation and de-activation or suppression at POD1 when compared to the baseline at POD0. This mixed profile is likely explained by simultaneous exposure to elevated levels of pro-inflammatory signals, including IL-6 and IL-1β, and counter-regulatory mediators such as IL-10 and cortisol (37, 38). However, this also differs for the distinct monocyte subsets. Immune activation can be delineated from increases of CD64 expression across all three monocyte subsets for day 1-3 after surgery, and increases in CD11b, CD14 and TLR4 expression in especially IM and CM. IM show similar changes as CM as well as an additional increase in CD64, CD62L expression.

In contrast, reduced expression of CD54, besides HLA-DR, as well as increased expression of glucocorticoid-sensitive alternative activation markers CD163, CD206 and CD121b can be interpreted as signs of monocyte de-activation or suppression. Overall, the HLA-DR phenotype in our study showed a rapid decline from POD0 to POD1 and then a trend toward preoperative values. At baseline (POD0) median fluorescence intensity of HLA-DR and CD54 is found higher in IM and NCM compared to CM, confirming previous findings by others (30). Kim et al. found HLA-DR to be negatively correlated with cortisol levels after vascular surgery, suggesting a mechanism for HLA-DR down-regulation (39). Of specific interest are the findings by Sint et al. (40), who observed that HLA-DR expression in monocytes is a predictor that was lower in patients who develop anastomotic leakage compared to those who did not develop anastomotic leakage.

### Limitations and strengths

It is important to note that individual variations between patients could significantly impact the leukocyte, and specifically the monocyte response. Therefore, a limitation to this study might be the relatively small number of patients. Another limitation might be the differences regarding sites of surgery. We have observed statistically significant differences in leukocyte responses depending on locations, but patient numbers are limiting robust conclusions in this regard. Furthermore, this study only considered colorectal surgery, which takes place in a non-sterile environment, and therefore direct comparisons with sterile surgery might show interesting differences at the level of circulating leukocytes. The rate of surgical site infections is for example substantially higher in patients undergoing colorectal surgery, with a current rate of 5% to 30% (41, 42),compared to an overall risk of 2% (43). Another limitation of the current analysis is that we have not distinguished between patients with an uncomplicated clinical trajectory, and those who developed postoperative complications. The latter occurred in 4 patients (8%) who were eventually diagnosed with anastomotic leakage after the time window of observation. With regard to the currently considered parameters, these patients did not immediately stand out from the uncomplicated patients. In a follow-up analysis of this cohort will address putative associations with postoperative complications.

As strengths of the study, we consider the inclusion of only treatment-naïve patients undergoing colorectal resections as part of cancer treatment with creation of an anastomosis. Thus, a homogeneous cohort has been compiled with narrow in- and exclusion criteria. Furthermore, consistency in sampling time and relatively short duration to analysis, as well as use of fresh samples are strengths of this study. However, the small numbers of patients call for a cautious interpretation.

### Final remarks

The current study gives insight into the immunological response after colorectal cancer surgery with creation of an anastomosis. We show that colorectal surgery gives large quantitative and qualitative shifts in leukocyte, and particularly monocyte subsets. To our knowledge this is the first prospective cohort study with in depth analysis of monocyte subsets and their phenotypic markers in relation to colorectal surgery. Future research may further investigate the predictive value of these parameters as putative predictors of early post-surgical complications.

## Data availability statement

The raw data supporting the conclusions of this article will be made available by the authors, without undue reservation.

## Ethics statement

The studies involving human participants were reviewed and approved by Erasmus Medical Center ethics committee. The patients/participants provided their written informed consent to participate in this study.

## Author contributions

CS, DL, PL, and JL planned the study. AO, NN, and WD collected samples and performed flow cytometry experiments. PE and PL wrote the manuscript draft. ND performed and supervised statistical analyses. EG, PC, AM, supervised the collection of clinical data and take responsibility for the integrity of the data. All authors were involved in drafting the article or revising it critically for important intellectual content, and all authors approved the final version to be published. All authors listed have made a substantial, direct and intellectual contribution to the work, and approved it for publication.

## Funding

This study was funded by Sofradim Production, a Medtronic plc company, having a place of business at 116 Avenue du Formans, Trevoux, France 01600.

## Acknowledgments

We would like to thank all patients who participated in this study. We are grateful to everyone in the department of surgery and laboratories of all participating hospitals: Havenziekenhuis, Rotterdam, the Netherlands, IJsselland Ziekenhuis, Capelle aan den IJssel, the Netherlands, Maasstad Ziekenhuis, Rotterdam. We also would like to thank our students Lisa de Jong, Kirsten de Groene and Lars van Greuningen for their valuable contributions to this project.

## Conflict of interest

The authors declare that the research was conducted in the absence of any commercial or financial relationships that could be construed as a potential conflict of interest.

## Publisher’s note

All claims expressed in this article are solely those of the authors and do not necessarily represent those of their affiliated organizations, or those of the publisher, the editors and the reviewers. Any product that may be evaluated in this article, or claim that may be made by its manufacturer, is not guaranteed or endorsed by the publisher.
