# Supplementary material for: Monocyte response after colorectal surgery: A prospective cohort study
Source: Front Immunol. 2022 Oct 27;13:1031216. doi: 10.3389/fimmu.2022.1031216 (PMC9647000; doi:10.3389/fimmu.2022.1031216)
Supplement: Supplementary file 1 [file DataSheet_1.docx]

### Suppl. Table 1: Antibody clones used in IMACS

| Marker | Label | Clone | Company | Cat. Nr. |
| --- | --- | --- | --- | --- |
| CD11b | AF488 (Fitc) | icrf44 | BD | 557701 |
| CD14 | APC-H7 | 61D3 | Invitrogen (Thermofisher) | 47-0149-42 |
| CD16 | PE-CY7 | ebioCB16 | Ebioscience | 25-0168-42 |
| CD45 | PO | HI30 | Invitrogen | MHCD4530 |
| CD54 | BV711 (PerCP) | HA58 | BD | 564078 |
| CD62L | FITC | FMC46 | BioRad  (AbD Serotec) | MCA1076F |
| CD64 | APC | 10.1 | BD | 561189 |
| CD66b | PB | G10F5 | BD | 562940 |
| CD121b (IL1) | PerCP | 34141 | Invitrogen (Thermofisher) | MA523661 |
| CD163 | PE | GHI/61 | BD | 556018 |
| CD206 | PerCP-Cy5.5 | 15-2 | Biolegend |  |
| HLA-DR | PE | LN3 | Invitrogen (Thermofisher) | 12-9956-42 |
| TLR4 | PE | TF901 | BD | 564215 |
| TLR5 | FITC | 85B152.5 | Invitrogen (Thermofisher) | MA5-16235 |

### Suppl. Fig. 1: Flow cytometry gating strategy


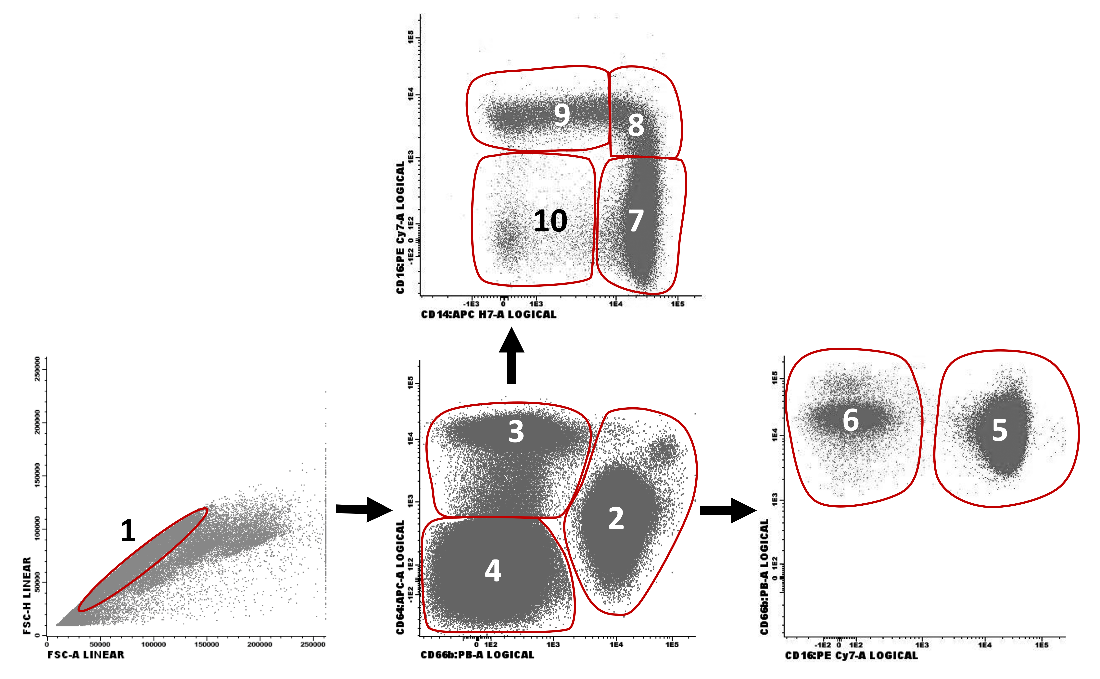


Leukocyte subpopulations were identified by flowcytometry as indicated. Briefly, singlet cells were gated (1), and then granulocytes (2), monocytes (3) and lymphocytes (4) were identified based on their differential expression of granulocyte marker CD66b and monocyte marker CD64. Granulocytes were further divided into neutrophils (5) and eosinophils (6) based on CD16 expression. Monocyte subsets were divided into CD14++16- classical monocytes (CM; 7), CD14++16+ intermediate monocytes (IM; 8), and CD14+16++ non-classical monocytes (NCM; 9). Finally, dendritic cells (DC; 10) were identified as CD14-16- cells.

**Suppl. Fig. 2: Monocyte subset immunophenotypic alterations after surgery**

| 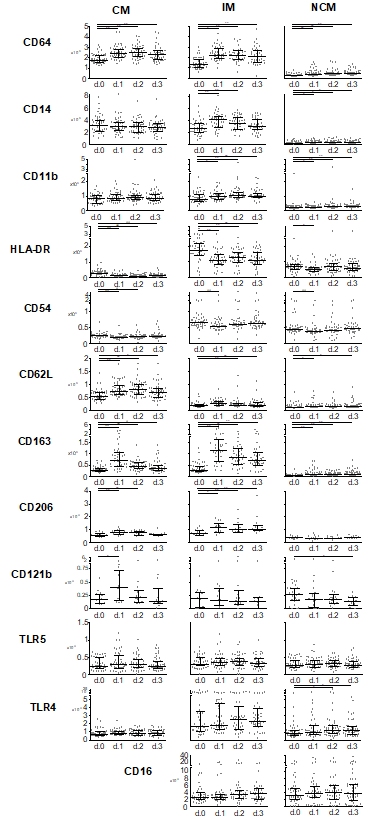 |
| --- |

### Suppl. Fig. 3: Significant correlations CM delta d.1-d.0 marker expression

| 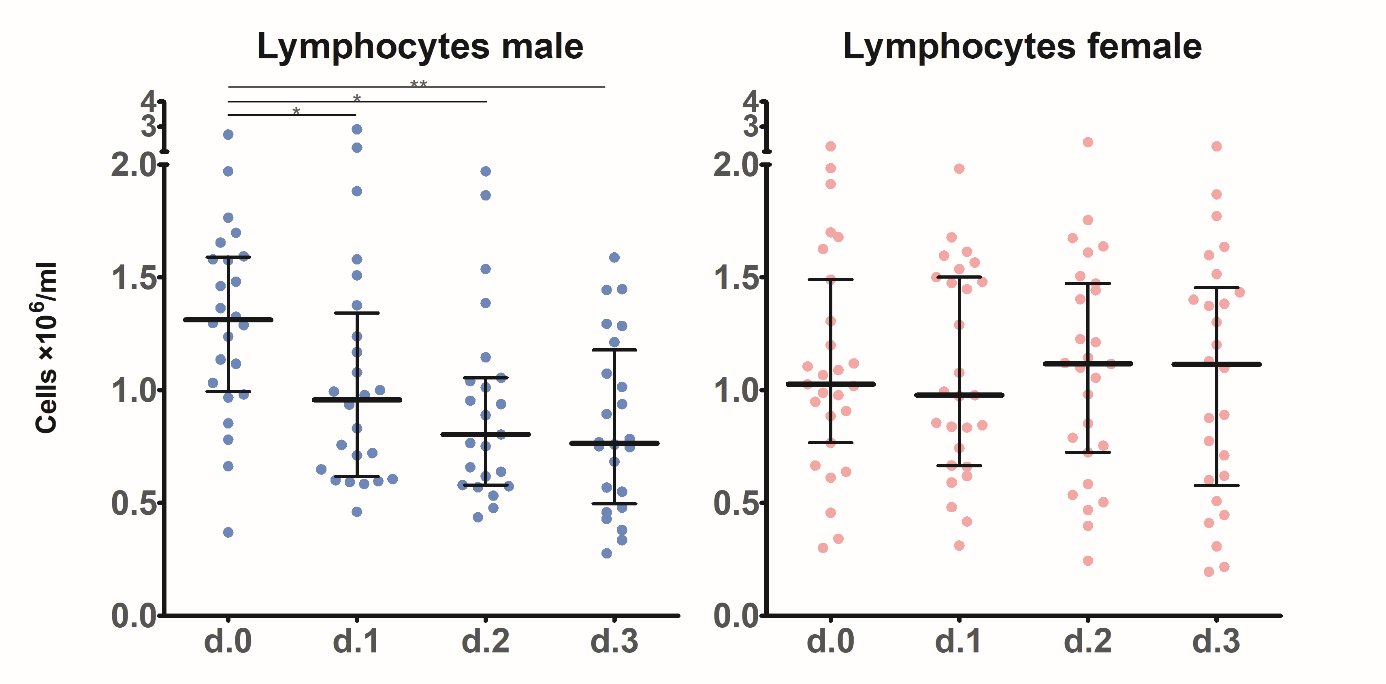  Absolute lymphocyte counts in circulation in male (left, in blue) vs. female (right, in pink) patients before and during the first three days after surgery as determined by flowcytometric immunophenotyping. Individual data points as well as medians and interquartile ranges are shown. Significant differences between d.0 and d.1, d.2 or d.3 are indicated: * p ≤ 0.05; ** 0.01 > p < 0.001. |
| --- |

### Suppl. Fig. 4: Significant correlations IM delta d.1-d.0 marker expression

| 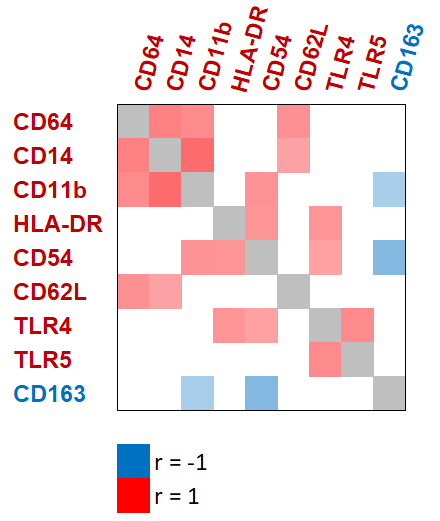 |
| --- |
